# MadR mediates acyl CoA-dependent regulation of mycolic acid desaturation in mycobacteria

**DOI:** 10.1073/pnas.2111059119

**Published:** 2022-02-14

**Authors:** Charlotte Cooper, Eliza J. R. Peterson, Rebeca Bailo, Min Pan, Albel Singh, Patrick Moynihan, Makoto Nakaya, Nagatoshi Fujiwara, Nitin Baliga, Apoorva Bhatt

**Affiliations:** ^a^School of Biosciences, University of Birmingham, Birmingham B15 2TT, UK;; ^b^Institute of Microbiology and Infection, University of Birmingham, Birmingham B15 2TT, UK;; ^c^Institute for Systems Biology, Seattle, WA 98109;; ^d^Otemae College of Nutrition, Osaka 599-8531, Japan;; ^e^Department of Food and Nutrition, Faculty of Contemporary Human Life Science, Tezukayama University, Nara 631-8585, Japan;; ^f^Department of Biology, University of Washington, Seattle, WA 98105;; ^g^Department of Microbiology, University of Washington, Seattle, WA 98105;; ^h^Molecular and Cellular Biology Program, University of Washington, Seattle, WA 98105;; ^i^Lawrence Berkeley National Lab, Berkeley, CA 94720

**Keywords:** *Mycobacterium*, tuberculosis, mycolic acid, TetR regulator, cell envelope

## Abstract

*Mycobacterium tuberculosis* has a lipid-rich cell envelope that is remodeled throughout infection to enable adaptation within the host. Few transcriptional regulators have been characterized that coordinate synthesis of mycolic acids, the major cell wall lipids of mycobacteria. Here, we show that the mycolic acid desaturase regulator (MadR), a transcriptional repressor of the mycolate desaturase genes *desA1* and *desA2*, controls mycolic acid desaturation and biosynthesis in response to cell envelope stress. A *madR*-null mutant of *M. smegmatis* exhibited traits of an impaired cell wall with an altered outer mycomembrane, accumulation of a desaturated α-mycolate, susceptibility to antimycobacterials, and cell surface disruption. Transcriptomic profiling showed that enriched lipid metabolism genes that were significantly down-regulated upon *madR* deletion included acyl-coenzyme A (aceyl-CoA) dehydrogenases, implicating it in the indirect control of β-oxidation pathways. Electromobility shift assays and binding affinities suggest a unique acyl-CoA pool–sensing mechanism, whereby MadR is able to bind a range of acyl-CoAs, including those with unsaturated as well as saturated acyl chains. MadR repression of *desA1*/*desA2* is relieved upon binding of saturated acyl-CoAs of chain length C_16_ to C_24_, while no impact is observed upon binding of shorter chain and unsaturated acyl-CoAs. We propose this mechanism of regulation as distinct to other mycolic acid and fatty acid synthesis regulators and place MadR as the key regulatory checkpoint that coordinates mycolic acid remodeling during infection in response to host-derived cell surface perturbation.

## Significance

Our studies show that the mycolic acid desaturase regulator (MadR) acts as a molecular switch, controlling the desaturation and biosynthesis of mycolic acids, key lipids of the cell envelopes of mycobacteria. MadR works by a distinct mechanism wherein it binds various acyl-coenzyme As (aceyl-CoAs), but only saturated acyl-CoAs relieve DNA binding and repression. This suggests a unique mechanism that involves sensing of acyl-CoA pools as a checkpoint for coordinating mycolic acid remodeling and biosynthesis in response to cell surface perturbation. Our findings further our understanding of how mycobacteria control cell wall composition in response to stress across various environments ranging from soil to an intracellular niche in infected macrophages, with implications for understanding strategies for pathogenesis in the tubercle bacillus.

The etiological agent of tuberculosis, *Mycobacterium tuberculosis*, has a distinct cell envelope among bacteria that influences its virulence and survival within the host. This novel cell envelope has been exploited as a drug target with the use of antitubercular agents, such as isoniazid (INH), thiolactomycin, and ethambutol (ETM) (1). The protective structure is largely composed of very long–chain fatty acids (up to C_90_) called mycolic acids (2). These are found functionally as free mycolic acids, as parts of the glycolipids, trehalose mono- and dimycolate, or covalently linked to the cell sacculus via esterification to the arabinogalactan–peptidoglycan complex. Mycolic acid biosynthesis is carried out by the two consecutive systems of fatty acid synthases I and II (FAS-I and FAS-II), responsible for generation of the α-chain and longer mero-chain, respectively. The multidomain FAS-I first coordinates the de novo synthesis of medium-chain–length fatty acyl-coenzyme As (acyl-CoAs) in a bimodal fashion (C_16_ to C_18_ and C_24_ to C_26_). The C_16_ to C_18_ CoAs feed into the FAS-II multienzyme complex, whereas the C_24_ to C_26_ CoAs go on to provide the α-chain of the mature mycolates. FAS-II is responsible for the successive rounds of elongation and modification required to generate the very long–chain (mero-chain) fatty acids that are the signature of mycobacterial species (2).

The bacterium’s ability to adapt the cell envelope composition in response to the varying conditions imposed by the host plays an important role in pathogenesis. However, studying mycolates and specific changes to their modifications and composition within the cell envelope during infection is particularly challenging (3). Although a number of landmark studies have revealed the global transcriptional regulatory network of *M. tuberculosis*, there have only been two regulators confirmed and characterized that control the expression of enzymes of mycolic acid synthesis (3–5). FasR regulates FAS-I by activation of the *fas-acpS* cluster, and MabR is an activator of the FAS-II gene cluster consisting of *fabD-acpM-kasA-kasB*, responsible for the biosynthesis of the longer mero-chain of mycolic acids (6, 7). Both transcription factors (TFs) have been shown to coordinate target promoter binding in response to long-chain acyl-CoA binding in vitro and upon uptake of exogenous fatty acids (1, 7, 8). Mycolic acids are not limited to pathogenic mycobacterial species, rather they are ubiquitous among all mycobacteria, including nonpathogenic environmental mycobacteria and also other related genera of the suborder Corynebacterineae (1). Thus, studying regulatory pathways that control mycolic acid biosynthesis could also shed light on the evolution of these regulatory mechanisms from a role in survival in environmental saprophytic species to bespoke roles in intracellular survival and dormancy in pathogenic species, such as *M. tuberculosis*.

**Fig. 1.**
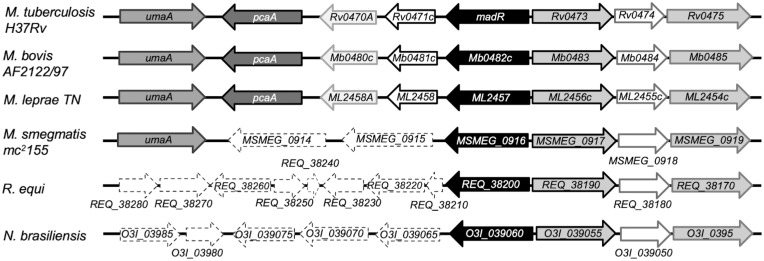
Gene maps of mycolate-producing actinobacteria with a MadR homolog. Homologous genes are shown from *M. bovis*, *M. leprae*, *M. smegmatis*, *Rhodococcus equi*, and *Nocardia brasiliensis*. Homologous genes are mapped with the same representative arrows; lack of homology of genes to *M. tuberculosis* is indicated by a white arrow with dashed borders.

We recently used a systems biology–driven approach to identify a TetR family TF in mycobacteria termed mycolic acid desaturase regulator (MadR), which is responsible for the regulation of the two aerobic mycolic acid desaturase genes *desA1* and *desA2* (3). While we initially identified the mycobacterial homologs of *M. tuberculosis* MadR (Rv0472c) (3), homologs of *madR* are found across all mycolic acid–producing genera (Fig. 1), suggesting an evolutionarily conserved role for MadR in mycolic acid–containing bacteria. The mero-chain double bonds introduced by DesA1 and DesA2 are a prerequisite for subsequent modifications, such as cyclopropanation, that are critical for virulence in *M. tuberculosis*. Loss of mycolic acid cyclopropanation leads to the complete attenuation of infection in mice (9–12). In murine alveolar macrophages, there is a co-ordinated up-regulation of *desA1* and *desA2* (4.0 and 4.7 log_2_ fold change, respectively) with *umaA* and *pcaA*, genes associated with mycolate cyclopropanation (2.6 and 1.5 log_2_ fold change). During in vitro infection of bone marrow–derived macrophages, *desA1*/*desA2* and *umaA* are similarly up-regulated during the 2 h of initial infection, returning to extracellular levels 8 to 24 h after infection (3). This suggests that, at least during initial infection, the cell wall mycolic acids are being remodeled to assist pathogenesis, with *desA1*/*desA2* driving this change cooperatively with cyclopropanation. The two desaturase genes are also essential components of the FAS-II mycolic acid biosynthesis machinery and thus are essential for survival (13, 14). Indeed, overexpression of *madR* in fast- and slow-growing mycobacteria led to bacterial cell death (3). Under anaerobic conditions inducing nonreplicative persistence (NRP), there was a transient increase in expression of *desA1*/*desA2* observed, followed by a significant repression (4.0 and 3.2 log_2_ fold change) after 40 h of hypoxia (15). In a corroborating study, *desA1* and *desA2* were found to be similarly up-regulated (4.7-fold and 2.5-fold) early in the transition to NRP and then repressed during stationary phase (16). Taken together, these implicate the regulation of *desA1*/*desA2* as having a dual role both responsible for driving the remodeling of mycolates early in infection (via modulating desaturation) and for limiting mycolate synthesis for persistence (by reducing overall mycolic acid biosynthesis via *desA1*/*desA2* repression).

Here, we investigated the consequences of deleting *madR* in the saprophytic species *M. smegmatis*, including its impact on the transcriptome of *M. smegmatis*. Furthermore, we queried the molecular mechanism of MadR-mediated repression of *desA1* and *desA2*.

## Results

### Loss of *madR* Affects *desA1* and *desA2* Expression in *M. smegmatis*.

**Fig. 2.**
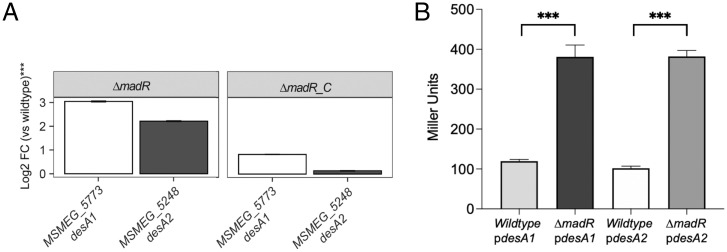
Deletion of *M. smegmatis madR* results in the up-regulation of *desA1* and *desA2*. (*A*) Bar graphs showing the log_2_ fold change (FC) expression in comparison to the WT of *desA1* and *desA2* for the sample sets *ΔmadR* and *ΔmadR_C* relative to the WT sample. Error bars show the 95% confidence interval. The asterisks (***) labeled in the *y* axis indicate statistical significance (*P* < 0.001) for both genes in all sample sets; *n* = 3 biological replicates. (*B*) β-galactosidase activities of *desA1* (p*desA1*) and *desA2* (p*desA2*) promoter *lacZ* transcriptional fusions in *M. smegmatis* WT and *ΔmadR* strains; ***, *P* < 0.001; *n* = 4 biological replicates. Error bars represent standard deviation (SD).

Using specialized transduction, we were able to generate a null mutant of *madR* in *M. smegmatis*, confirming that the gene encoding this regulator of key mycolic acid biosynthesis genes was not essential for growth in laboratory media. We also did not see any obvious differences in growth rate of the *M. smegmatis madR* mutant (*SI Appendix*, Fig. S1). We then performed RNA sequencing of the *madR* mutant along with the parental wild type (WT) and complemented strain (Dataset S1). Both *desA1* and *desA2* were significantly up-regulated (*P* < 0.01) in *M. smegmatis* Δ*madR* compared to WT by 3.0 and 2.2 log_2_ fold change, respectively, while they were not significantly differentially expressed in the complemented strain compared to WT (Fig. 2*A* and *SI Appendix*, Table S2). Their genomic neighbors were also significantly up-regulated, indicating that *desA1* (*MSMEG_5773*) is cotranscribed with *MSMEG_5774*, and *desA2* (*MSMEG_5248*) is cotranscribed with *MSMEG_5247*. The operon partner with *desA2*, *MSMEG_5247*, is a probable PhoH family protein, indicating activity during phosphate starvation, but is otherwise uncharacterized. Likewise, the operon partner of *desA1*, *MSMEG_5774*, is uncharacterized, and the functional association of these genes with the mycolate desaturases is unknown, if any. In addition to *desA1*, *desA2*, and their operon partners, 31 other genes were significantly induced in Δ*madR* cells compared to WT and complement (*SI Appendix*, Table S2). Interestingly, genes adjacent to *madR* were not differentially expressed, which is common for regulators of the TetR family (17), although exceptions are known (18). Comparing the significantly induced genes to the predicted *M. tuberculosis* MadR regulon (*Rv0456A*, *desA1*, *desA2*, *fdxC*, *Rv1178*, *tgs1*, and *Rv3131*) derived through both physical binding (from chromatin immunoprecipitation with sequencing [ChIP-seq] experiments [4]) and functional evidence (from transcriptional profiling [5]) found that *desA1*, *desA2*, and *Rv3131* were conserved MadR targets in both species. This suggests that MadR controls a small regulon of nonadjacent genes involved in lipid metabolism. Among the 84 genes that were significantly down-regulated in Δ*madR* cells compared to WT and complement, there were significant functional term clusters (*P* < 0.01) defined by Database for Annotation, Visualization and Integrated Discovery (DAVID) (19) related to transmembrane transport and lipid metabolism. More specifically, the enriched lipid metabolism genes that were significantly down-regulated upon *madR* deletion were genes encoding putative acyl-CoA dehydrogenases (*MSMEG_0323*, *MSMEG_2650*, *MSMEG_5045*, *MSMEG_6585*, and *MSMEG_6686*). This may suggest that fatty acid β-oxidation is reduced in Δ*madR* cells, although mycobacterial genomes encode multiple enzymes for each step of β-oxidation (20), including other putative acyl-CoA dehydrogenases that were not differentially expressed in Δ*madR* cells (*SI Appendix*, Fig. S2).

We were also able to independently demonstrate the effects of *madR* deletion on *desA1* and *desA2* expression using recombinant LacZ reporter plasmids, where *lacZ* expression was driven by either the *desA1* or *desA2* promoter. Transformants of Δ*madR* containing these constructs exhibited fourfold higher LacZ activity than transformants of the WT strain containing the same plasmids. Together, these data confirmed that the in vivo loss of *madR* results in the up-regulation of *desA1* and *desA2* (Fig. 2*B*).

### Deletion of *madR* in *M. smegmatis* Alters the Proportion of Desaturated α-mycolic Acid Species by Dysregulation of *desA1* and *desA2*.

**Fig. 3.**
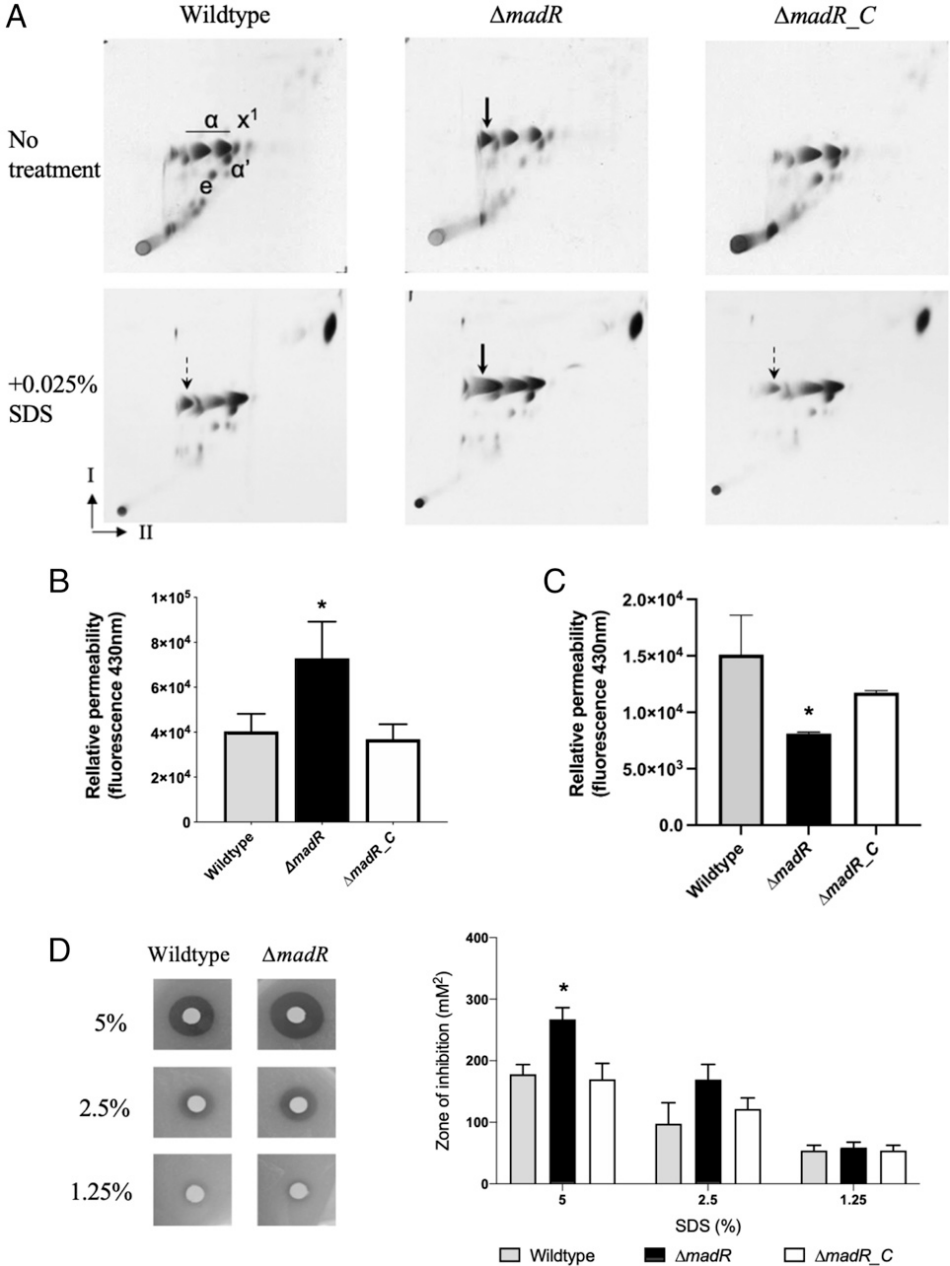
Deletion of *M. smegmatis madR* results in accumulation of a hyperdesaturated α-MAME, altered outer mycomembrane, and susceptibility to cell surface disruption. (*A*) Two-dimensional argentation TLC lipid analysis from [^14^C]-acetic acid–labeled *M. smegmatis* WT, Δ*madR*, and Δ*madR_C* whole-cell fatty acid methy esters (FAMEs) and MAMEs untreated or treated with 0.025% SDS. Fractions were loaded at 15,000 cpm onto silica TLC plates and run in direction I with 2× petroleum ether 60–80/acetone (19:1 [vol/vol]) and direction II with 3× petroleum ether 60–80/ethyl acetate (18:2 [vol/vol]); e, epoxy-MAMEs; α and α′, α-MAME subclasses; x_1_, cyclopropanated α-MAME derivative. A solid arrow denotes the position of the hyperdesaturated α-MAME as a result of *madR* deletion; a dashed arrow indicates the position of the hyperdesaturated α-MAME in the WT and complement strains as a result of cell surface disruption. TLCs are representative of *n* = 2 biological replicates. (*B*) Relative permeability of *M. smegmatis* WT, Δ*madR*, and Δ*madR_C* strains as measured by DPH fluorescence at 430 nm. Error bars represent SD; *, *P* < 0.05; *n* = 6 biological replicates. (*C*) DPH binding to the extracted mAGP complex of strains as measured by fluorescence at 430 nm. Error bars represent SD; *, *P* < 0.05; *n* = 3 biological replicates. (*D*) Sensitivity as measured by zone of inhibition of WT, *ΔmadR*, and *ΔmadR_C* strains subjected to treatment with 5%, 2%, and 1.25% SDS by disk diffusion assay; *, = *P* < 0.05; *n* = 6 biological replicates. Error bars represent SD.

As MadR represses *desA1* and *desA2* expression, we next sought to determine whether dysregulation of these desaturase genes impacted mycolic acid profiles (3). Methyl esters of ^14^C-labeled mycolic acids (MAMEs) were extracted from cells of *M. smegmatis* WT, Δ*madR*, and Δ*madR_*C strains and analyzed by two-dimensional (2D) argentation thin layer chromatography (TLC). Silver nitrate impregnated into the second dimension provides separation based on level of unsaturation, with higher levels of unsaturation corresponding to increased retardation in this dimension. Loss of *madR* resulted in relatively higher abundance of an α-MAME subspecies that showed a high degree of retardation in the silver-containing dimension (at the base of the AgNO_3_ phase; arrow in Fig. 3*A*, *Top*) suggesting that it had a high degree of desaturation. This “highly” desaturated α*-*MAME was also present in the WT strain, albeit at a much lower abundance than other α*-*MAME subspecies. These patterns were consistent across external, cell wall–bound, and internal apolar lipid fractions (*SI Appendix*, Fig. S4). MAME profiles were restored to those seen in WT in the complemented strain, indicating that the observed changes were solely due to loss of *madR* (Fig. 3*A*). We further characterized MAMEs extracted from unlabeled cells by high performance liquid chromatography (HPLC)-electrospray ionization (ESI)/mass spectrometry (MS) (*SI Appendix*, Fig. S4). The results revealed no qualitative differences between the profiles of the WT and mutant strains (i.e., no unique species in the mutant strain); however, MAME species with *m/z* values of 1,120 (α-C74:1) and 1,148 (α-C76:1) were relatively more abundant in the Δ*madR* mutant than in the WT strain (*SI Appendix*, Fig. S4).

### Deletion of *madR* Results in an Altered Outer Mycobacterial Membrane.

We reckoned that the altered α-MAME patterns may disrupt the structure of the cell envelope, preventing it from being able to assemble as uniformly and thus may increase the cell envelope permeability. To probe this further, we used permeation by *trans*-1,6-diphenyl-1,3,5-hexatriene (DPH) fluorescence as a way of assessing potential changes in cell wall permeability. As a hydrophobic hydrocarbon similar in size to the length of a phospholipid acyl chain, DPH is able to insert into the cell membrane and align to the acyl chains of lipids within the layer (21–23). The *madR*-null mutant exhibited significantly higher incorporation of DPH than the WT and complement strains (Fig. 3*B*). We explored the possibility that this increased incorporation of DPH may also be a result of differential binding to the mycobacterial “outer membrane”, primarily the mycolic acid layer bound to the peptidoglycan–arabinogalactan complex. We extracted the mycolyl-arabinogalactan–peptidoglycan (mAGP) sacculus from WT, Δ*madR*, and Δ*madR_*C strains and measured DPH incorporation in the extract. Surprisingly, mAGP from Δ*madR* showed lower incorporation of DPH than mAGP from WT or Δ*madR_*C strains. These results suggested that DPH was able to better access the inner membrane of the Δ*madR* strain. While we cannot conclusively show that the *madR* mutant had increased cell wall permeability, our findings suggest an altered cell envelope in the mutant.

A number of deletion mutants with impaired cell wall synthesis and cell envelope defects also show increased susceptibility to lipophilic antibiotics (24–28). Lipophilic drugs, such as rifampicin (RIF), penetrate into the cell by passive diffusion through the hydrophobic cell wall components, whereas uptake of hydrophilic drugs, such as INH and ETM, must be facilitated through porins embedded in the cell wall. Therefore, cell envelope defects or alterations may result in increased susceptibility to the lipophilic antibiotic RIF (29). Minimum inhibitory concentrations (MICs) for RIF, ETM, and INH were determined by resazurin microtiter assay. The *M. smegmatis madR*-null mutant exhibited four times greater sensitivity to RIF with a MIC of 1 µg/mL than the WT and complemented strains at 4 µg/mL. Interestingly, Δ*madR* also showed increased sensitivity to INH (4 µg/mL) compared to the WT and complemented strains (8 µg/mL). In WT *M. tuberculosis*, *desA1*/*desA2* are significantly down-regulated in response to 0.18 µg/mL INH for 4, 24, and 72 h (at 24 h, log_2_ fold change = −2.50 and −2.712 for *desA1* and *desA2*, respectively; *P* < 0.01), and, therefore, the inability of MadR to repress expression may account for this increased susceptibility in the deletion strain (30, 31). There was no change in the MIC for ETM between the strains.

### MadR Responds to Cell Envelope Stress to Remodel Cell Envelope Mycolic Acids.

Expression data for *M. tuberculosis* indicated that *desA1* is up-regulated in response to cell wall perturbation by the detergent sodium dodecyl sulfate (SDS) (32). This is interesting considering the up-regulation of *desA1* observed here, as the loss of *madR* in *M. smegmatis* results in a higher proportion of desaturated mycolates and, consequently, increased cell envelope permeability. Low cell membrane permeability and high rigidity are important countermeasures against cell wall–disruptive agents in other bacteria. This is often modulated by the ratios of saturated and unsaturated fatty acids within a membrane (33, 34). We performed disk diffusion assays with a range of concentrations of SDS and scored susceptibility as a measure of the zone of inhibition. The Δ*madR* deletion strain was significantly more susceptible to cell envelope stress by treatment with 5% SDS than the WT and complemented strains (Fig. 3*C*).

Exploring this susceptibility in the context of mycolates, strains were subjected to treatment with 0.025% SDS, and ^14^C-labeled MAMEs were extracted from whole cells before analysis by 2D argentation TLC. There was no difference observed in *madR* MAMEs between the untreated and SDS-treated extracts; we saw the same abundance of the “highly” unsaturated α-MAME species in the Δ*madR* extracts from both Δ*madR*-treated and untreated cells. However, interestingly, this altered abundance was now seen in SDS-treated WT and Δ*madR*_C strains (shown by the arrow in Fig. 3*A*, *Bottom*). This suggested that *madR* in the WT strain relieves repression of *desA1* and *desA2* in response to cell surface disruption, mirroring the mycolate profile we observed in the untreated Δ*madR*-null mutant. However, as *madR* in the WT strain is still present to act as a regulator, the abundance of this α-MAME is somewhat more limited.

RNA sequencing of strains subjected to 0.025% SDS treatment was carried out to explore this on a transcriptional level (Dataset S1). Following SDS treatment, *desA1* was significantly up-regulated by 1.3 log_2_ fold change versus WT (not SDS treated); however, *desA2* was only modestly up-regulated by 0.5 log_2_ fold change (*SI Appendix*, Fig. S5*A*). The gene encoding the fatty acid desaturase DesA3 (*MSMEG_1886*) was significantly up-regulated following SDS treatment (log_2_ fold change = 1.75) but not upon *madR* deletion and could be more important for desaturation events than DesA2 during SDS treatment.

The same LacZ reporter strains described earlier (Fig. 2*B*) were now used to probe these transcriptional observations for *desA1* and *desA2* upon treatment with SDS. β-galactosidase activities of WT cells containing the *desA1* or *desA2* promoter–*lacZ* fusions were significantly increased upon treatment with 0.025% SDS (*SI Appendix*, Fig. S5*B*). However, in the *ΔmadR* strain, no differences were observed between SDS-treated and nontreated cells, indicating a MadR-driven differential expression (DE) of *desA1* and *desA2* in response to the cell envelope stress (*SI Appendix*, Fig. S5).

### MadR Exists as a Dimer and Binds to the Promoters of *desA1*/*desA2.*

To further study the mechanisms of MadR-mediated repression of *desA1* and *desA2* expression, we first purified *M. tuberculosis* MadR (Rv0472c) following heterologous expression in *Escherichia coli.* MadR is a predicted TetR-like transcriptional repressor, and members of this family of TFs oligomerize via the C-terminal domain to function as a dimer and bind to target promoter DNA via the N-terminal domain (35). Analysis by SDS-polyacrylamide gel electrophoresis (PAGE) indicated that recombinantly expressed *M. tuberculosis* MadR is a protein of 26 kDa, consistent with the predicted molecular weight based on the amino acid sequence (*SI Appendix*, Fig. S6*A*). Gel filtration revealed that *M. tuberculosis* MadR elutes at a volume corresponding to a dimer (52 kDa), confirming that MadR exists as a dimer in solution (*SI Appendix*, Fig. S6*B*).

**Fig. 4.**
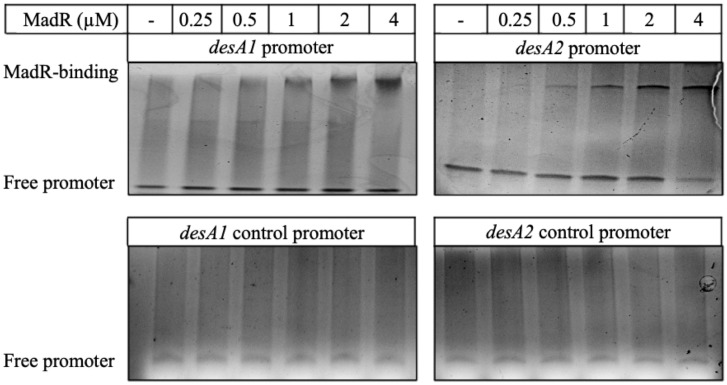
*M. tuberculosis* MadR binds the promoters of *desA1* and *desA2*. Native PAGE results of the electromobility shift assay for MadR at a range of concentrations binding to the *desA1* and *desA2* promoter regions and corresponding control oligos. Gels were stained with a nucleic acid dye for visualization of double-stranded DNA (dsDNA). Bands corresponding to free promoter and MadR-bound promoter are indicated; gels are representative of *n* = 2 replicates.

Next, to demonstrate in vitro *M. tuberculosis* MadR binding to the promoters of *M. tuberculosis desA1* and *desA2,* electromobility shift assays were performed using oligos designed around the respective ChIP-seq binding peaks identified by Minch et al. (4). Alignment of the ChIP-seq peak regions of *desA1* and *desA2* showed a “TCTGTGxxxxxxxGT” consensus in the promoters. Binding of *M. tuberculosis* MadR to both the *desA1* and *desA2* promoter fragments was observed by a protein concentration–dependent shift of the promoter DNA on native PAGE (Fig. 4, *Top*). No gel shifts were observed with control oligos that included substitutions in the binding motif (Fig. 4, *Bottom*) or with the unrelated *hsp60* promoter region (*SI Appendix*, Fig. S7), ruling out nonspecific binding of MadR to DNA.

### Effect of Acyl-CoAs on *M. tuberculosis* MadR Binding to Target Promoters.

Previously characterized transcriptional regulators of mycolic acid biosynthesis act in response to acyl-CoA ligands (1, 7), suggesting that biosynthesis is unsurprisingly co-ordinated with fatty acyl metabolism, primarily products of degradation pathways (β-oxidation), but also FAS-I mediated biosynthesis. Indeed, β-oxidation of fatty acids released from triacly glycerol (TAGs) can be channeled to mycolic acid biosynthesis (36). We found that the predicted structure of MadR is close to that of DesT, the regulator of the fatty acid desaturase genes (*desBC*) in *Pseudomonas aeruginosa* (1, 37, 38). DesT differentially regulates the expression of the *desBC* operon by distinguishing between saturated and unsaturated long-chain acyl-CoAs (37). To test for interactions between *M. tuberculosis* MadR and potential acyl-CoA ligands, we chose a series of physiologically relevant acyl-CoAs and their fatty acid equivalents with varying chain lengths and levels of desaturation (C_16:0_-CoA, C_16:1_-CoA, C_18:0_-CoA, C_18:1_-CoA, C_18:2_-CoA, C_18:3_-CoA, C_20:0_-CoA, C_22:0_-CoA, and C_24:0_-CoA) and assessed their binding to MadR.

Quantification of binding affinity for the acyl-CoA species was performed by measurement of intrinsic tryptophan fluorescence quenching. Interestingly, MadR was able to bind all of the acyl-CoA species tested but to varying affinities (*SI Appendix*, Fig. S8 *A* and *B*). Binding affinities peaked with stearoyl-CoA (C_18:0_-CoA), exhibiting the highest affinity for MadR at 7.73 µM, and ranged to the lowest with lignoceroyl-CoA (C_24:0_) at 18.15 µM. The saturated acyl-CoAs followed the trend that the further the chain length increases, or decreases, from C_18_, the more the binding affinity declines. For example, binding affinities changed from 7.73 µM for C_18:0_-CoA to 10.68 µM for palmitoyl-CoA (C_16:0_-CoA) and 12.43 µM for myristoyl-CoA (C_14:0_-CoA) (*SI Appendix*, Fig. S8*A*). A similar pattern was observable in the case of unsaturated acyl-CoA species, wherein the higher the degree of desaturation in the acyl chain, the lower the affinity was for MadR (*SI Appendix*, Fig. S8*B*). This was exemplified by oleoyl-CoA (C_18:1_), linoleoyl-CoA (C_18:2_), and linolenoyl-CoA (C_18:3_) that exhibited binding affinities of 9.55 µM, 9.88 µM, and 11.59 µM, respectively.

**Fig. 5.**
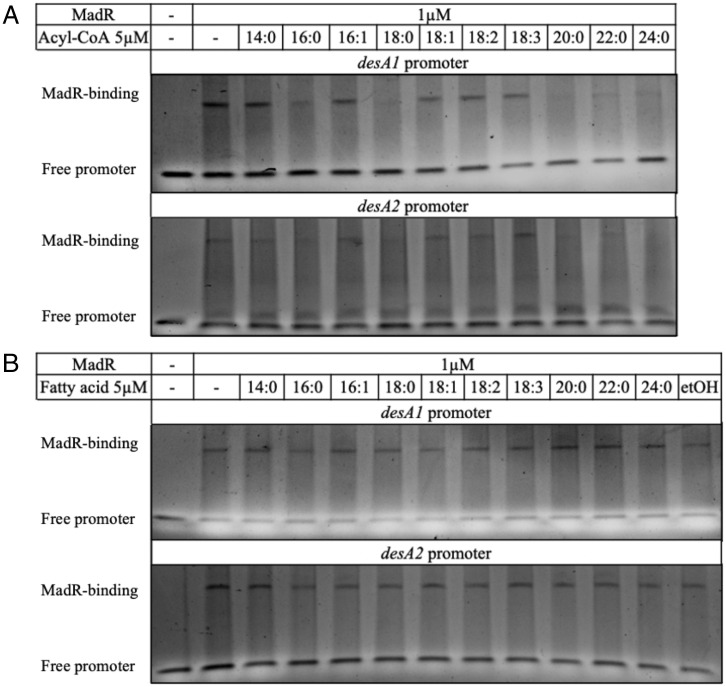
*M. tuberculosis* MadR responds to saturated acyl-CoAs ≥C_16_ to relieve binding on the promoters of *desA1* and *desA2* in a CoA-dependent manner. (*A*) Native PAGE results of the electromobility shift assay for MadR at 1 µM binding to the desA1 and desA2 promoter regions in the presence of 5 μM C_16:0_-CoA, C_16:1_-CoA, C_18:0_-CoA, C_18:1_-CoA, C_18:2_-CoA, C_18:3_-CoA, C_20:0_-CoA, C_22:0_-CoA, and C_24:0_-CoA. (*B*) Native PAGE results of the electromobility shift assay for MadR at 1 µM binding to the *desA1* and *desA2* promoter regions in the presence of 5 μM C_16:0_, C_16:1_, C_18:0_, C_18:1_, C_18:2_, C_18:3_, C_20:0_, C_22:0_, and C_24:0_ fatty acids. Gels were stained with a nucleic acid dye for visualization of dsDNA. Bands corresponding to free promoter and MadR-bound promoter are indicated; gels are representative of *n* = 2 replicates.

Next, we tested the ability of *M. tuberculosis* MadR to bind promoter DNA in the presence of fatty acids and fatty acyl-CoAs. Saturated acyl-CoAs with a chain length of ≥C_16_ released binding of MadR to both *M. tuberculosis desA1* and *desA2* promoter regions (Fig. 5*A*). In contrast, unsaturated acyl-CoAs did not affect promoter binding (Fig. 5*A*). These results also appear to be CoA dependent, as there was no difference in the promoter shift observed in assays performed with the fatty acid equivalents (Fig. 5*B*). Long-chain fatty acids and fatty acyl-CoAs can act as detergents at high concentration and cause protein denaturation (7). The lack of difference in gel shift seen with the addition of fatty acids compared to the control mitigates for this detergent effect. Therefore, the loss of gel shifts with the saturated long-chain acyl-CoAs are a result of release of MadR binding rather than denaturation. Fatty acids were solubilized in ethanol, and so a control lane for this was included; this did not interfere with MadR binding to the promoter regions.

Despite the ability to bind to all the acyl-CoA species assayed, only saturated acyl-CoA species with chain lengths ≥C_16_ were able to release binding of MadR to the *desA1*/*desA2* promoters. These results suggest a mechanism whereby MadR senses the acyl-CoA pool to modulate *desA1*/*desA2* expression in response to increases in saturated fatty acyl-CoAs, fine tuning expression to substrate supply.

## Discussion

The results here inform a picture of MadR as a regulator of mycolic acid biosynthesis, specifically the desaturation step. DesA1 and DesA2 are hypothesized to work on an elongating mero-chain and as part of a FAS-II complex (14). Thus, their absence also shuts down mycolic acid biosynthesis. Surprisingly, mycolic acid profiles of the *madR* mutant matched those of the WT strain treated with SDS to mimic cell envelope stress. The *madR* mutant was also more sensitive to SDS treatment, suggesting a role for MadR in regulating mycolate desaturation following cell envelope damage or stress. Indeed, loss of *madR* led to higher levels of *desA1* and *desA2* expression. Our in vitro studies with purified MadR showed that MadR binding to *desA1*/*desA2* promoters is relieved by fatty acyl CoAs but not fatty acids. More specifically, saturated acyl-CoAs (≥C_16_) relieved MadR binding to the promoter, while unsaturated acyl-CoAs did not, thus suggesting that while MadR binds both saturated and unsaturated acyl-CoAs, effects on promoter binding are specific. Further work on the molecular mechanisms of MadR binding to ligand and promoter elements should shed light on how (and whether) competition between saturated and unsaturated acyl-CoAs for MadR has consequences for mycolic acid desaturation and biosynthesis. The binding of MadR is mechanistically different to other regulators of bacterial fatty acid desaturases, such as FabR of *E. coli* and closely related DesT of *P. aeruginosa*, which bind to both unsaturated and saturated acyl-CoA and elicit a DNA recognition response to both, either inducing/enhancing or relieving repression to the target promoters (37, 39, 40). In contrast, MadR is able to bind a range of acyl-CoAs regardless of the level of desaturation, but only upon binding saturated acyl-CoAs of C_16_ chain length and above is binding of these promoters relieved. These chain lengths are particularly significant because FAS-II exclusively uses C_16_- to C_24_-chain–length CoAs for elongation into the mero-mycolic acid chain. Although FAS-I is able to synthesize C_16_ to C_24_ fatty acyl-CoAs, the predominant species generated are C_16_- and C_18_-CoA (36). This directly reflects the binding affinities observed in this work, with MadR displaying the highest affinity for C_16_- and C_18_-CoAs, with chain lengths deviating away from these experiencing lower binding affinities.

The transcriptional activator of the FAS-II operon MabR similarly binds to long-chain acyl-CoAs between C_18_ and C_24_ to activate expression of the FAS-II operon members (*fabD-acpM-kasA-kasB-accD6*) (1, 6). Additionally, FasR is the regulatory activator of the *fasI-acpS* operon and responds to long-chain acyl-CoAs >C_16_-CoA to inhibit DNA binding and transcriptional activation (7, 8). This presents a regulatory system wherein FAS-I expression is down-regulated in response to its output (C_16_- to C_24_–CoA), and both FAS-II operon members and *desA1*/*desA2* are up-regulated in response to their long-chain acyl-CoA substrates (C_18_- to C_24_–CoA and C_16_- to C_24_–CoA, respectively). Distinctively, MadR is able to bind a range of fatty acyl-CoAs regardless of chain lengths and unsaturation levels. The ability of MadR to sense the acyl-CoA pool in this way uniquely distinguishes this regulatory mechanism from the other known mycolate transcriptional regulators and may constitute a distinct regulatory “checkpoint” in mycolate biosynthesis. Mycobacteria are able to synthesize mycolic acids in the absence of FAS-I activity, with the primers for mero-chain elongation provided by β-oxidation of fatty acids released from triacylated glycerols (36). These degradation intermediates would be acylated CoAs and may possibly act as ligands of MadR. Interestingly, RNA-sequencing analysis showed a down-regulation of genes encoding putative acyl-CoA dehydrogenases in the *madR* mutant, suggesting an indirect role for MadR in the control of bespoke β-oxidation pathways and by extension the composition of acyl-CoA pools in the mycobacterial cell.

We have demonstrated here that disruption of this regulatory mechanism by deletion of *madR* results in the accumulation of a highly desaturated α-mycolate species. This mycolate species is consistent with expression data wherein the loss of MadR regulation on *desA1*/*desA2* results in significant up-regulation of these desaturases. Therefore, there is a concomitant increase in desaturation events on the mycolate chain. Consequently, the deletion mutant exhibits increased cell wall permeability, susceptibility to RIF and INH and susceptibility to cell surface disruption by SDS. Upon SDS treatment, the WT strain shows an accumulation of the same highly desaturated mycolate species and a significant up-regulation of *desA1* and *desA2*, albeit to a lesser extent. This is indicative of MadR relieving repression of these targets in response to cell wall perturbation.

**Fig. 6.**
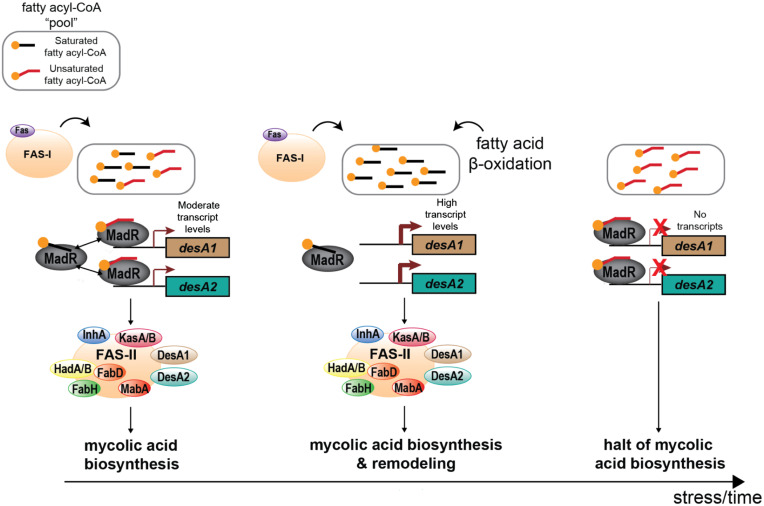
Proposed mechanism of MadR regulation of mycolic acid desaturation. During normal growth under laboratory conditions, desaturation of mycolic acids is coordinated by MadR regulation of *desA1* and *desA2* by sensing the supply of >C_16_ saturated fatty acyl-CoA output from FAS-I. Dominance of unsaturated acyl-CoAs or lack of saturated acyl-CoA in the cellular pool due to reduced FAS-I output leads to *desA1*/*desA2* repression by *madR* and shutdown of mycolic acid synthesis. Under cell surface disruption, a shift in the acyl-CoA pool from β-oxidation of exogenous or endogenous fatty acid sources leads to *madR* relieving repression of *desA1*/*desA2*, resulting in mycolate and cell wall remodeling. In *M. tuberculosis*, nonreplicative persistence likely induces a lack of saturated acyl-CoA or build-up of unsaturated acyl-CoA, leading to the consistent repression of *desA1*/*desA2* and shutdown of mycolic acid synthesis.

Here, we present a unique mechanism of regulation among the known mycolic acid TFs, wherein MadR is able to sense the composition of the fatty acyl-CoA pool by its ability to bind to fatty acyl-CoAs regardless of the level of unsaturation or chain length (Fig. 6). Under normal growth conditions, MadR is able to fine-tune expression of *desA1*/*desA2* in response to the availability of saturated fatty acyl-CoAs ≥C_16_, mycolic acid synthesis precursors generated by FAS-I. The absence of these precursors, or build-up of unsaturated fatty acyl-CoA, leads to the repression of *desA1*/*desA2*, resulting in the reduction of mycolic acid biosynthesis. However, under cell surface disruption, MadR functions to relieve repression on *desA1*/*desA2*, likely in response to the availability of saturated fatty acyl-CoA supplied from β-oxidation of endogenous or exogenous fatty acid sources that may accumulate under this stress condition. Both *desA1* and *desA2* are down-regulated drastically in *M. tuberculosis* after 40 h of hypoxia-induced dormancy (3). We can speculate that under these conditions, there is an absence of saturated acyl-CoA, or build-up of unsaturated acyl-CoA, from the uncoupling of FAS-I from FAS-II to redirect saturated acyl-CoA precursors into TAG biosynthesis (41). MadR, therefore, may respond to repress its desaturase targets and reduce the levels of mycolic acid biosynthesis. Thus, MadR represents a regulatory checkpoint for mycolic acid synthesis in response to metabolic cues. Furthermore, given the presence of homologs of MadR in other mycolic acid–producing species, it is tempting to speculate that MadR represents an ancestral regulator of mycolic acid biosynthesis with evolved functions for common and unique roles in environmental and pathogenic mycolate-producing genera. The ability of MadR to repress desaturase gene expression (and by extension mycolate biosynthesis) could, with a further understanding of the molecular mechanisms of MadR regulation, highlight its potential for exploitation as a drug target, in this case, the development of ligand-mimicking compounds that confer an “always bound” state to MadR, effectively shutting off the essential process of mycolic acid biosynthesis.

## Materials and Methods

### Culture Conditions.

*Mycobacterium smegmatis* was cultured aerobically in Middlebrook 7H9 supplemented with 10% oleic acid-albumin-dextrose-catalse (OADC) supplement, 0.2% glycerol, and 0.05% Tween-80 at 37 °C. *M. smegmatis* strains were maintained with the addition of 50 μg/mL hygromycin B and 25 μg/mL kanamycin where appropriate. *E. coli* strains used for cloning and knockout generation were cultured in Luria broth (LB) and supplemented with 150 μg/mL hygromycin and 50 μg/mL kanamycin where appropriate. Disk diffusion assays were performed as previously described with 6-mm Whatman filter disks soaked with 10 µL of 5%, 2.5%, and 1.25% SDS (42). Plates were counted after 24 h of incubation at 37 °C.

### Deletion Strain Generation.

Approximately 1-kb gene-flanking regions of *M. smegmatis madR* (*MSMEG_0916*) were PCR amplified from *M. smegmatis* mc^2^155 genomic DNA (gDNA) with the primers *MSMEG0916*_AlwNI_RL, *MSMEG0916*_AlwNI_RR, *MSMEG0916*_AlwNI_LR, and *MSMEG0916*_AlwNI_LL (*SI Appendix*, Table S1). Direct cloning of the AlwNI-digested PCR fragments and digested p0004s were transformed into *E. coli* Top10 cells. Recovered sequence-positive constructs were linearized with PacI and ligated with phAE159 DNA in *E. coli* HB101 cells for phasmid generation. The resultant phasmid was packaged with MaxPlax Lambda Packaging Extracts (Lucigen) to generate the knockout phage phΔ*MSMEG0916* designed to replace the chromosomal copy of *madR* with a hygromycin resistance cassette. High titers of phΔ*MSMEG0916* were generated in *M. smegmatis* at the permissive temperature of 30 °C and used to generate a *madR*-null mutant by specialized transduction, as described previously (43). Gene replacement in hygromycin-resistant transductants was confirmed by whole-genome sequencing, and one strain designated *M. smegmatis* Δ*madR* was used for all subsequent studies.

### Construction of Complemented Strains.

*M. smegmatis madR* (*MSMEG_0916*) with flanking promoter and terminator sites was PCR amplified for cloning into the integrative vector pMV306 (44) using the primers pMV306_*MSMEG0916*_F and pMV306_*MSMEG0916*_R (*SI Appendix*, Table S1). Sequence-positive constructs were electroporated into *M. smegmatis* Δ*madR* and selected for using 25 μg/mL kanamycin, as previously described (45). Complemented strains were confirmed by PCR of gDNA to confirm integration, and one strain was selected for subsequent analysis and is referred to as *M. smegmatis* Δ*madR_C*.

### Determination of Antibiotic MIC.

Aerobic resazurin reduction assays were performed for determining MIC values. MIC was considered the lowest drug concentration with an inhibition of >90%. Percentage inhibition was calculated to be 1 − (fluorescence/mean positive-control fluorescence) × 100 (46). Briefly, ∼5 × 10^4^ cells were incubated at 37 °C in 7H9 supplemented with 10% OADC and 0.2% glycerol in the presence of a twofold dilution series of RIF, ETM, or INH for 24 h. After incubation, 30 μL of 0.02% resazurin and 25 μL of 10% Tween-80 were added to wells and allowed to incubate for a further 3 h at 37 °C. Fluorescence readings were obtained using an excitation wavelength of 530 nm and an emission wavelength of 590 nm using a PHERAstar FS microtiter plate reader (BMG Labtech). MIC assays were performed in triplicate.

### DPH Binding Assays.

Relative cell wall permeability was assessed using permeation by DPH (21). Strains were cultured to mid-log phase before harvesting and fixing with 0.25% formaldehyde for 1 h at room temperature. Cells were washed with phosphate-buffered saline (PBS), resuspended in PBS supplemented with 2.5 μM DPH, and incubated for 30 min at 37 °C in the dark. Fluorescence was measured using a PHERAstar FS microtiter plate reader with an emission wavelength of 430 nm and an excitation wavelength of 358 nm. Relative permeability is a measure of DPH fluorescence in comparison to fluorescence of untreated control wells. Isolation of mAGP was carried out using methods described previously (47), and DPH binding was carried out using the same protocol as above using mAGP pellets instead of cells. Assays were performed at least in triplicate.

### Labeling, Extraction, and Analysis of Mycolic Acids.

Radiolabeling with [^14^C]-acetic acid (PerkinElmer) (1 mCi/mL) was performed on cultures at mid-log phase (optical density at 600 nm (OD_600_) = 0.5) for 24 h before harvesting or after 30 min of treatment with or without 0.025% SDS and harvested after a 6-h incubation at 37 °C. Mycolic acids were extracted using a modified method based on the method described in ref. 48. Dried whole-cell pellets were subjected to alkaline hydrolysis by 10% tetrabutylammonium hydroxide overnight at 100 °C and methylation by addition of 4 mL of CH_2_Cl_2_, 500 µL of CH_3_I, and 2 mL of water for 30 min. The resultant lower organic phase was collected and washed with water to recover fatty acid and MAMEs. For analysis by 2D argentation TLC, samples were loaded at 15,000 counts per minute (cpm) onto Silica Gel 60 F254 plates and resolved, unless otherwise stated, using petroleum ether 60–80/acetone (19:1 [vol/vol]) twice in the first direction and three times in petroleum ether 60–80/ethyl acetate (18:2 [vol/vol]) in the second direction. Autoradiograms (Carestream Kodak BioMax MR) were exposed for 3 d.

### Mass Spectrometry.

ESI/MS was performed on a 4000 QTRAP LC-MS/MS System (SCIEX Corp.) with an Acquity UPLC H-class-Bio (Waters Corp.). In liquid chromatography/mass spectrometry experiments, an XTerra MS C18 column (125 Å, 3.5 µm, 2.1 mm × 150 mm; Waters Corp.) was utilized for separation of MAMEs. Methanol (mobile phase A) and chloroform (mobile phase B) were used for gradient elution. Initial conditions were mobile phase at 90% A/10% B followed by a linear gradient to 10% A/90% B in 40 min. The IonSpray voltage was maintained at 4.2 kV. The temperature was 600 °C for MAMEs. The column eluent was introduced into the Turbo Spray ion source of an ESI/MS system operated in positive ion mode. The mass spectra were acquired from *m/z* 700 to 1,500 with a frequency of 1 scan/0.90 s for MAMEs. Typically, 10 μL of sample was injected for analysis. Analyst 1.6.2 software (SCIEX Corp.) was used for system control, data accumulation, and data analysis.

### RNA Isolation.

Strains were cultured to mid-log phase (OD_600_ = 0.5) in Middlebrook 7H9 supplemented with 10% OADC, 0.2% glycerol, and 0.05% Tween-80 at 37 °C. Cultures were left untreated or treated with 0.025% SDS for 90 min at 37 °C, harvested, and snap-frozen in liquid nitrogen before RNA isolation. The resuspended cells were transferred to a tube containing Lysing Matrix B (MP Biomedicals) and vigorously shaken at max speed for 30 s in a FastPrep 120 homogenizer (MP Biomedicals) three times. Tubes were centrifuged for 1 min (max speed), and 66 μL of 3 M sodium acetate, pH 5.2, was added and mixed well. Acid phenol (pH 4.2) was added at 726 μL, and tubes were inverted to mix well (∼60 times). Samples were incubated at 65 °C for 5 min, inverting tubes to mix samples every 30 s, and centrifuged at 14,000 rpm for 5 min; the upper aqueous phase was transferred to a new tube. Sodium acetate (pH 5.2, 3 M) was added at 1/10th volume along with 3× volumes of 100% ethanol. The sample was mixed well and incubated at −20 °C for 1 h or overnight. Following incubation, samples were centrifuged at 14,000 rpm for 30 min at 4 °C, ethanol was discarded, and 500 μL of 70% ethanol was added. Samples were centrifuged again at 14,000 rpm for 10 min at 4 °C, supernatant was discarded, and any residual ethanol was removed using a pipet. The pellet was allowed to air dry, resuspended in 30 to 40 μL of RNase-free water, and quantified by Nanodrop (Thermo Scientific). This was followed by in-solution gDNA digestion using RQ1 DNase (Promega), following the manufacturer’s recommendations. RNA quality was analyzed in a 2100 Bioanalyzer system (Agilent Technologies). Total RNA (1 μg) was depleted of ribosomal RNA (rRNA) using the Ribo-Zero Bacteria rRNA Removal kit (Illumina).

### Processing and Analysis of RNA-Sequencing Data.

Quality and purity of messenger RNA (mRNA) samples was determined with a 2100 Bioanalyzer (Agilent). Samples were prepared with a TrueSeq Stranded mRNA HT library preparation kit (Illumina) and multiplexed into a single run. All samples were sequenced on a NextSeq sequencing instrument in a high-output 150 v2 flow cell. Paired-end 75-bp reads were checked for technical artifacts using Illumina default quality-filtering steps. Raw FASTQ read data were processed using the R package DuffyNGS, as described previously (49). Briefly, raw reads were filtered for rRNA transcripts and aligned against the *M. smegmatis* mc^2^155 genome (ASM1500v1) with Bowtie2 (50) using the command line option “very-sensitive”. BAM files recorded both uniquely mapped and multiply mapped reads to each of the forward and reverse strands of the genome(s) at single-nucleotide resolution. Across the 18 samples, the average mapped reads were 4,963,668 with a minimum of 3,043,651 and a maximum of 6,627,450. Gene transcript abundance was then measured by summing total reads landing inside annotated gene boundaries, expressed as both reads per kilobase of transcript per million mapped reads (RPKM) and raw read counts. Two stringencies of gene abundance were provided using all aligned reads and by just counting uniquely aligned reads. The raw and processed RNA-sequencing data generated for this study are available in the Gene Expression Omnibus under accession number GSE171759.

### DE.

We used a panel of five DE tools to identify gene expression changes with respect to WT *M. smegmatis* (in the absence of SDS treatment). The tools included 1) RoundRobin (in-house), 2) RankProduct (51), 3) significance analysis of microarrays (SAM) (52), 4) EdgeR (53), and 5) DESeq2 (54). Each DE tool was called with appropriate default parameters and operated on the same set of transcription results, using RPKM abundance units for RoundRobin, RankProduct, and SAM and raw read count abundance units for DESeq2 and EdgeR. All five DE results were then synthesized by combining gene DE rank positions across all five DE tools. Specifically, a gene’s rank position in all five results was averaged, using a generalized mean to the 1/2 power, to yield the gene’s final net rank position. Each DE tool’s explicit measurements of DE (fold change) and significance (*P* value) were similarly combined via appropriate averaging (arithmetic and geometric mean, respectively). Genes with an averaged absolute log_2_ fold change larger than 1 and a multiple hypothesis–adjusted *P* value below 0.01 were considered differentially expressed.

### Protein Expression and Purification.

The primers pET28a_*Rv0472c*_F and pET28a_*Rv0472c*_R (*SI Appendix*, Table S1) were used to PCR amplify *M. tuberculosis madR* (*Rv0472c*) from *M. tuberculosis H37Rv* gDNA for cloning into the pET28a expression vector. Sequence-confirmed pET28a_*Rv0472c* was cotransformed with *M. tuberculosis* GroES 60.2 chaperone (pTrc_GroES 60.2) (55) by heat shock into *E. coli* BL21(DE3) cells and selected for on LB supplemented with kanamycin (50 µg/mL) and ampicillin (100 µg/mL). Expression cultures were grown to an OD_600_ of 0.5 to 6 at 37 °C in terrific broth supplemented with appropriate antibiotics. Induction with 0.5 mM isopropyl β-d-1-thiogalactopyranoside was carried out overnight at 16 °C. Cells were harvested and frozen at −80 °C for storage. Cells were suspended in buffer 1 (20 mM Tris, pH 7.8, 300 mM NaCl, and 10% glycerol supplemented with ethylenediaminetetraacetic acid [EDTA]-free protease inhibitor [Roche]). Cells were lysed by sonication (MSE Soniprep 150), and the clarified supernatant was applied to cobalt-IMAC (immobilized metal affinity chromatography) resin (HisPur cobalt resin, Thermo Fisher Scientific), followed by washing with increasing imidazole concentrations in buffer 1. Fractions were collected, and buffer exchange was performed into buffer 1 before concentration supplemented with 1 mM dithiothreitol (DTT) by centrifugation and snap-freezing for storage at −80 °C. Purified protein was applied to a Superdex 200 pg HiLoad 26/600 column in buffer 1 supplemented with 1 mM DTT for determination of oligomeric state.

### Electromobility Shift Assays.

Oligos of the promoter regions of *desA1* and *desA2* were designed as 50 bp surrounding the ChIP-seq peak of *M. tuberculosis* MadR binding (4): *desA1*_P_−19/+31_F, *desA1*_ P_−19/+31_R, *desA2*_ P_−36/+14_F, and *desA2_* P_−36/+14_R. Control oligos were generated by substituting conserved bases within the promoter region: *desA1*_P_−19/+31_F_C, *desA1*_ P_−19/+31_R_C, *desA2*_ P_−36/+14_F_C, and *desA2_* P_−36/+14_R_C (*SI Appendix*, Table S1). Oligos were resuspended in annealing buffer (10 mM Tris, pH 8.0, 50 mM NaCl, and 1 mM EDTA), mixed in equimolar volume, heated to 95 °C for 2 min, and cooled to 25 °C over 45 min before being stored at −20 °C. The intergenic region of *hsp60* was used as a control and amplified with the primers *hsp60*_P_F and *hsp60*_P_R (*SI Appendix*, Table S1).

Purified MadR was concentrated to 1 mg/mL and snap-frozen at −80 °C for storage. Immediately prior to the assay, the protein was diluted to the desired concentration in binding buffer (20 mM Tris, pH 6.8, 50 mM NaCl, 1 mM DTT, and 1 mM EDTA). The reaction mixture consisted of 50 µg/mL bovine serum albumin, 50 µg/mL sonicated salmon sperm DNA, and 100 ng of promoter and appropriate concentration of MadR in a 10-µL reaction volume. After addition of protein, the reaction was allowed to proceed for 30 min at 22 °C. Samples were mixed with 5 µL of 6× Gel Loading Dye, no SDS (NEB), loaded onto mini-PROTEAN TGX 12% precast gels (Bio-Rad), and run at 100 V for 1.5 h at 4 °C in 1× Tris-glycine–EDTA buffer (20 mM Tris, pH 8.0, 150 mM glycine, and 1 mM EDTA). The gels were stained with Midori Green Advance (NIPPON Genetics) nucleic acid stain for 45 min at room temperature before visualization under ultraviolet transillumination. Ligand-binding gel shift assays were performed under the same conditions with the addition of myristoyl-CoA (C_14:0_-CoA), palmitoyl-CoA (C_16:0_-CoA), palmitoleoyl-CoA (C_16:1_-CoA), stearoyl-CoA (C_18:0_-CoA), oleoyl-CoA (C_18:1_-CoA), linoleoyl-CoA (C_18:2_-CoA), linolenoyl-CoA (C_18:3_-CoA), arachidonoyl-CoA (C_20:0_-CoA), behenoyl-CoA (C_22:0_-CoA), and lignoceroyl-CoA (C_24:0_-CoA) and their respective fatty acid equivalents to a final concentration of 5 μM.

### Intrinsic Tryptophan Fluorescence Quenching.

Intrinsic tryptophan fluorescence quenching was used to quantify acyl-CoA binding affinities by fluorescence spectroscopy. Acyl-CoAs were titrated from stock solution into 10 μM purified protein buffer 1. Protein fluorescence was measured at an excitation of 280 nM and an emission of 340 nM, with slits set at 5 nM (Hitachi F7000). Curve correction was performed for normalization of acyl-CoA fluorescence and fitted to the binding equation *Y* = *B*_max_ × *X*(*K*_d_ + *X*), where *Y* is the fluorescence of MadR, and *X* is the concentration of the acyl-CoA species. Binding assays were performed in triplicate.

### LacZ Transcriptional Fusions.

Regions ∼300 bp upstream and 100 bp downstream of the *desA1*/*desA2* start sites were amplified using the primers *desA1*_P_XbaI, *desA1*_P_SphI, *desA2*_P_XbaI, and *desA2*_P_SphI (*SI Appendix*, Table S1) and subsequently cloned into the promoterless *lacZ* construct pSD5B. The empty vector, *desA1*, and *desA2* transcriptional fusion constructs were electroporated into WT *M. smegmatis* and *M. smegmatis ΔmadR* and selected for on tryotic soy broth agar supplemented with 25 µg/mL kanamycin and 50 µg/mL X-gal. Strains were cultured to an OD_600_ of 0.5 in 7H9 supplemented with 0.2% glycerol and 25 µg/mL kanamycin, and β-galactosidase assays were performed in six replicates, as previously described (56).

## Supplementary Material

Supplementary File

Supplementary File

## Data Availability

All study data are included in the article and/or supporting information.
