# Supplementary material for: MadR mediates acyl CoA-dependent regulation of mycolic acid desaturation in mycobacteria
Source: Proc Natl Acad Sci U S A. 2022 Feb 14;119(8):e2111059119. doi: 10.1073/pnas.2111059119 (PMC8872791; doi:10.1073/pnas.2111059119)
Supplement: Supplementary File [file pnas.2111059119.sapp.pdf]

PNAS
www.pnas.org

Supplementary Information for

MadR mediates acyl CoA-dependent regulation of mycolic acid desaturation in mycobacteria

Charlotte Cooper[1], Eliza J. R. Peterson[2], Rebeca Bailo[1], Min Pan[2], Albel Singh[1], Patrick Moynihan[1], Makoto Nakaya[3], Nagatoshi Fujiwara[4], Nitin Baliga[2, 5, 6, 7*], Apoorva Bhatt[1*]

[1]School of Biosciences and Institute of Microbiology and Infection, University of Birmingham, Birmingham, UK
[2]Institute for Systems Biology, Seattle, WA, USA
[3]Otemae College of Nutrition, Osaka, Japan
[4] Department of Food and Nutrition, Division of Contemporary Human Life Science, Tezukayama University, Nara, Japan
[5]Departments of Biology and Microbiology, University of Washington, Seattle, WA, USA
[6]Molecular and Cellular Biology Program, University of Washington, Seattle, WA, USA
[7]Lawrence Berkeley National Lab, Berkeley, CA, USA

* Apoorva Bhatt, Nitin Baliga
Email: a.bhatt@bham.ac.uk, nitin.baliga@isbscience.org

This PDF file includes:

Figures S1 to S8
Tables S1 to S2

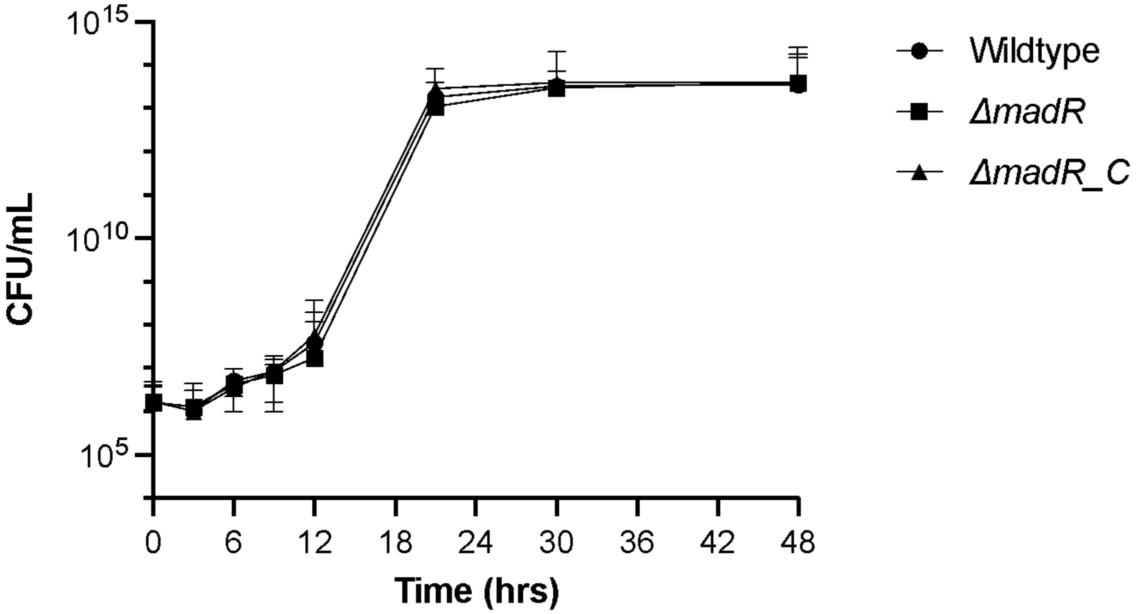

Fig. S1. Growth characteristics of the M. smegmatis madR deletion mutant in liquid culture. *Growth curves of wildtype, ΔmadR and madR_C strains as determined by CFU enumeration. Error bars represent standard deviation, n=3 biological replicates.*

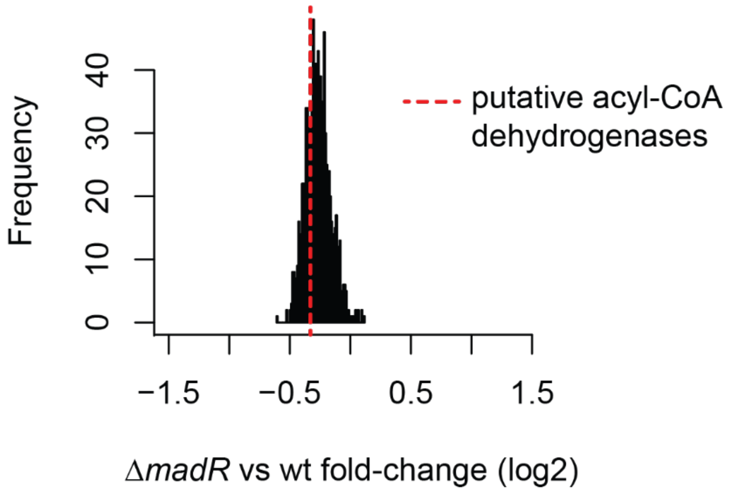

Fig S2. Expression of acyl-CoA dehydrogenases in ΔmadR strain vs WT. *Histogram of mean fold-change between ΔmadR strain vs WT from 1000 permutations to generate shuffled gene sets. In each permutation, the produced shuffled gene set had the same size as M. smegmatis homologs of putative acyl-CoA dehydrogenases encoded in M. tuberculosis (27 genes). The red line represents the observed value for the putative acyl-CoA dehydrogenases.*

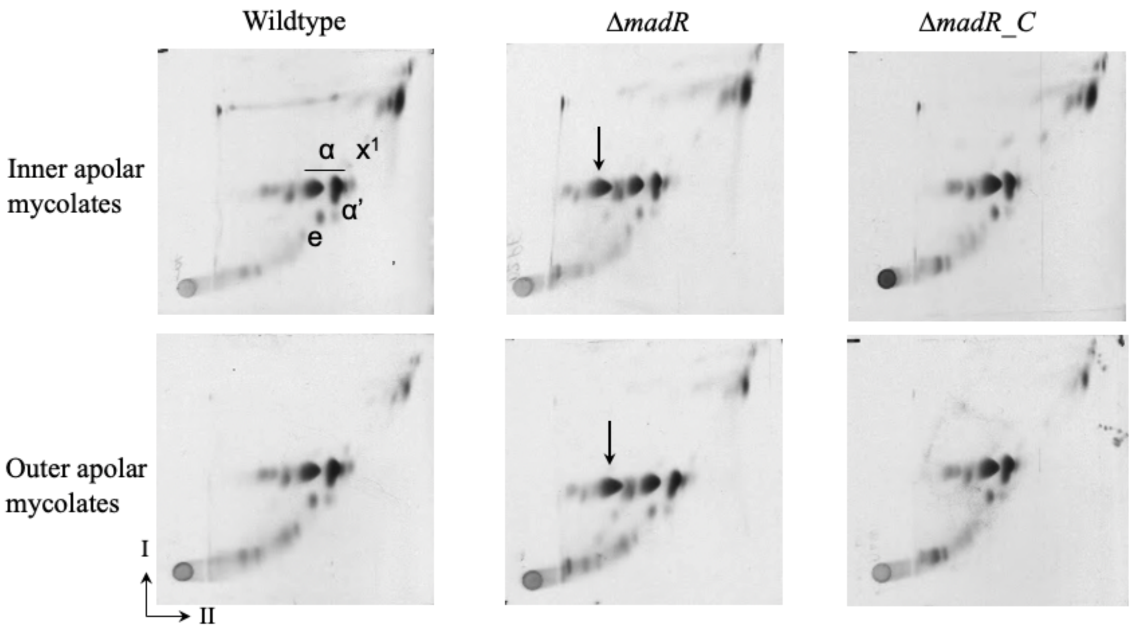

Fig S3. The highly desaturated mycolate associated with madR deletion in M. smegmatis is found in both the inner free mycolate and outer cell wall bound mycolate fractions. *2D-argentation TLC lipid analysis from [^{14}C]- acetic acid labelled M. smegmatis wildtype, ΔmadR and ΔmadR_C FAMEs and MAMEs extracted from the inner and outer apolar fractions. Fractions were loaded at 15,000cpm onto silica TLC plates and run direction I with 2x petroleum ether 60-80/acetone (19:1, v/v) and direction II with 3x petroleum ether 60-80/ethyl acetate (18:2, v/v). e = epoxy-MAMEs; α and α’ = α-MAME subclasses; x_1 = cycloproponated α-MAME derivative. An arrow denotes the position of the highly desaturated α-MAME as a result of MSMEG_0916 deletion, TLCs representative of n=2 biological replicates.*

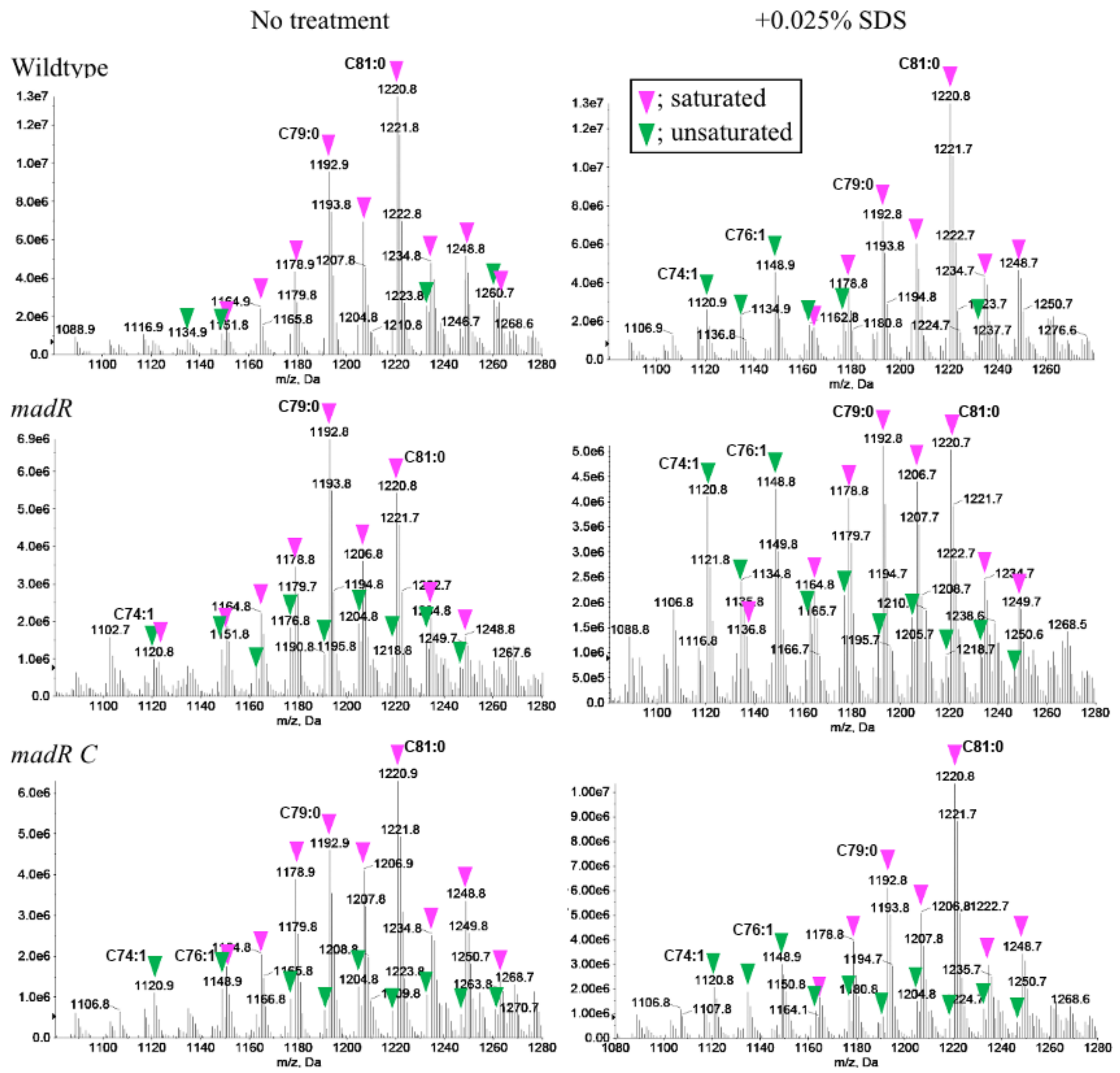

Fig S4. *HPLC-ESI/MS performed on a 4000 QTRAP LC-MS/MS system with an Acquity UPLC H-class-Bio. The separation of MAMEs was by a Xterra MS C18 Column. Methanol (mobile phase A) and chloroform (mobile phase B) were used for gradient elution; the mobile phase at 90% A/10% B followed by a linear gradient to 10% A/90% B in 40 min. The ESI/MS was operated in positive ion mode. The mass spectrum was acquired from m/z 700-1,500 with a frequency of 1 scan/0.90 s for MAMEs. The MAME peaks were indicated by inverted triangle for saturation; pink, unsaturation; green.*

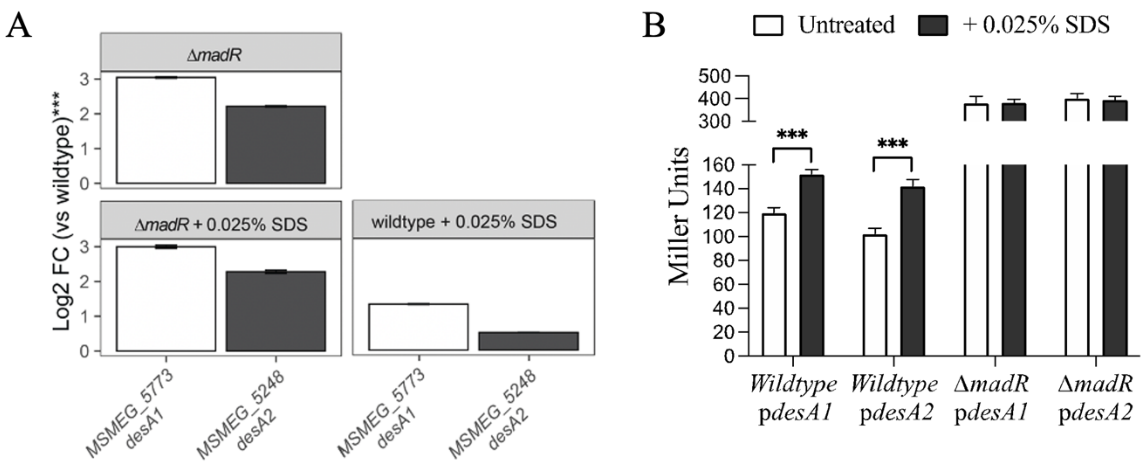

Fig S5. *A) Bar graphs showing the log2 fold change (FC) expression of desA1 and desA2 for each of the sample sets (∆madR, ∆madR +0.025% SDS, wildtype + 0.25% SDS) relative to wildtype sample. Error bars show the 95% confidence interval. *** labeled in the y-axis indicates statistical significance (p<0.001) for both genes in all sample sets, n=3 biological replicates. B) ß-galactosidase activities of desA1 (pdesA1) and desA2 (pdesA2) promoter LacZ transcriptional fusions in M. smegmatis wildtype and ∆madR strains untreated or treated with 0.025% SDS, ***= p<0.001, n=4 biological replicates. Error bars represent standard deviation.*

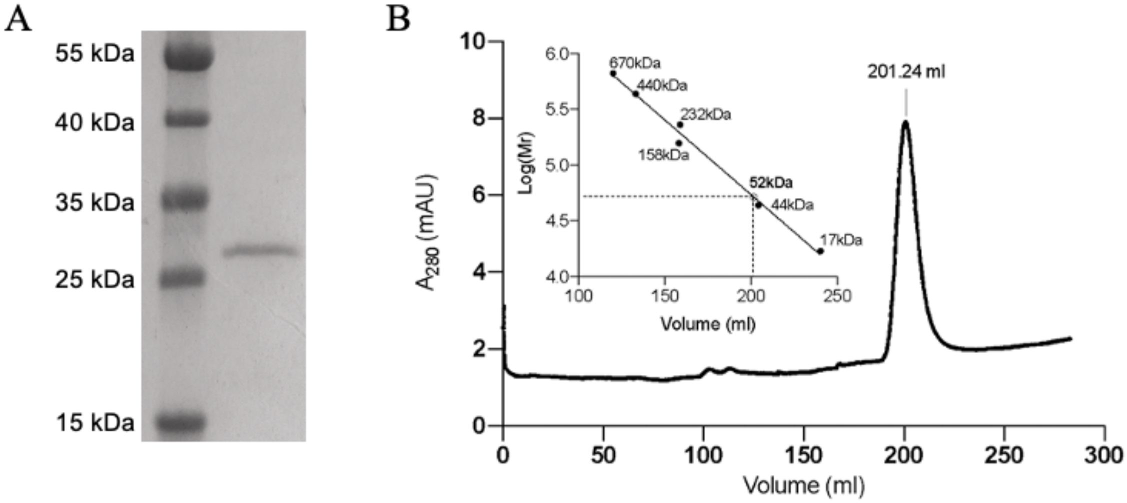

Fig S6. Purified recombinantly expressed M. tuberculosis MadR exists as a dimer in solution. *A) SDS-PAGE analysis indicating MadR monomeric molecular weight of 26kDa B) Gel filtration elution profile of MadR demonstrates that it elutes at a volume corresponding to a molecular weight of 52kDa as determined by comparison to known molecular weight standards and indicative of MadR existing as a dimer in solution, n=1.*

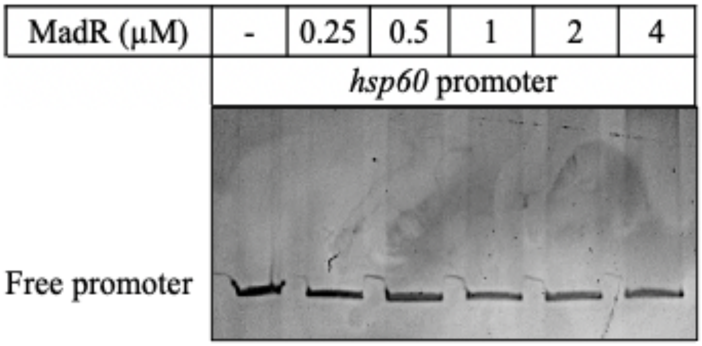

Fig S7. *Native-PAGE results of electromobility shift assay for MadR at differing concentrations with the unrelated hsp60 control promoter**.** Gels were stained with a nucleic acid dye for visualization of dsDNA. Bands corresponding to free promoter are indicated, Gel representative of n=2 biological replicates.*

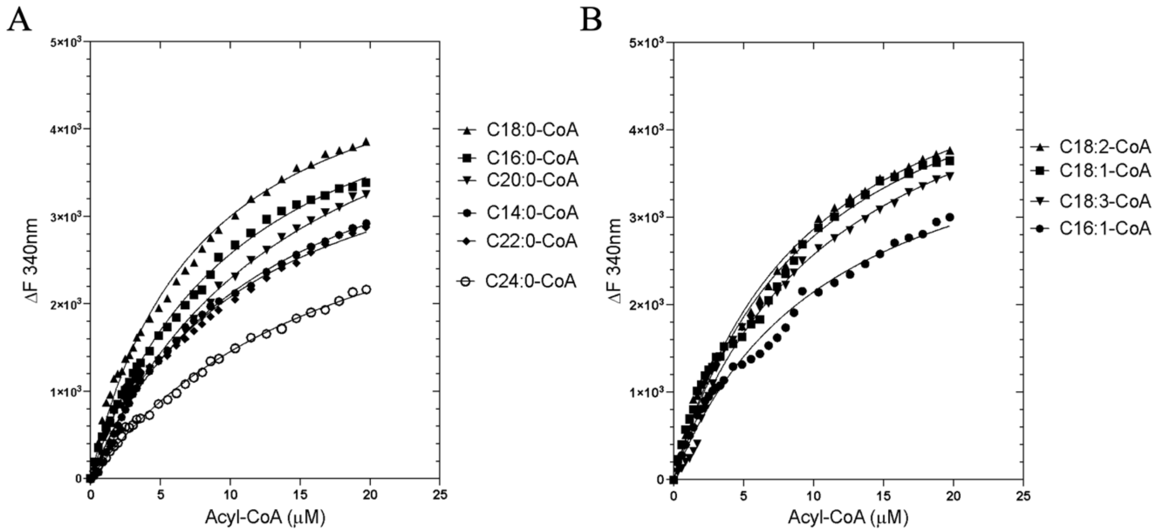

Fig S8. Tryptophan fluorescence quenching of acyl-CoAs for M. tuberculosis MadR. *Fluorescence quenching of MadR at 10 µM by A) saturated and B) unsaturated acyl-CoAs. Acyl-CoAs were titrated from stock solution and fluorescence measured at excitation 280nm and emission 340nm, n=3 biological replicates.*

Table S1. Oligos, sequences and for which function they were used in this study.

Name	Sequence	Function
*MSMEG0916*_AlwNI_RL	5'-TTTTTTTTCAGAGACTGGACGAGCTGA TCGACTAC-3'	*madR* AES substrate for knockout generation
*MSMEG0916*_AlwNI_RR	5'- TTTTTTTTCAGCTTCTGGCCTCGACATCG AAATCC-3'	*madR* AES substrate for knockout generation
*MSMEG0916*_AlwNI_LR	5'- TTTTTTTTCAGTTCCTGCGGTGGTGAGAT CGTTCTT-3'	*madR* AES substrate for knockout generation
*MSMEG0916*_AlwNI_LL	5'- TTTTTTTTCAGAAACTGAATCGCGTTCGA TGACCC-3'	*madR* AES substrate for knockout generation
pMV306_*MSMEG0916*_F	5'-TTTTCTAGAGACGAAGGTTCCCAGCC-3'	*ΔmadR* complement strain generation
pMV306_*MSMEG0916*_R	5'-TTTAAGCTTTTCGAACCGCGAAAGCC-3'	*ΔmadR* complement strain generation
pET28a_*Rv0472c*_F	5'-TTTTTTAAGCTTCTAGACCTGCCCCCAG-3'	Protein expression
pET28a_*Rv0472c*_R	5'- TTTTTTGGATCCGTGGCAGAGCGTATCCC-3'	Protein expression
pVV16_*Rv0472c*_F	5' -TTTTTGGATCCCTAGACCTGCCCCCAG -3'	Ligand pulldown
pVV16_*Rv0472c*_R	5'- TTTTAGCTTGTGGCAGAGCGTATCCC -3'	Ligand pulldown
*desA1*_P_-19/+31_F	5'-CAGTTATCTGTGTCTAATGGTTGC AGTCAATATATCTGTGTCTCTCGGTA-3'	EMSA
*desA1*_ P_-19/+31_R	5'-TACCGAGAGACACAGATATATTGACT GCAACCATTAGACACAGATAACTG-3'	EMSA
*desA2*_ P_-36/+14_F	5'-TTCACGAAATCTGTGTATGCGAGT TACAGTTACCGCATGGCACAGAAACC-3'	EMSA
*desA2*_ P_-36/+14_R	5'-GGTTTCTGTGCCATGCGGTAACTG TAACTCGCATACACAGATTTCGTGAA-3'	EMSA
*desA1*_P_-19/+31_F_C	5'- CCGTTCGAGACGACTAATGAGGTG CAACAATATACGATCACCATCGGTA-3'	EMSA
*desA1*_ P_-19/+31_R_C	5'- TACCGATGGTGATCGTATATTGTTG CACCTCATTAGTCGTCTCGAACGG-3'	EMSA
*desA2*_ P_-36/+14_F_C	5'- TTCGAGAACGATGCAAATACGAAG GTATACTACCTCATGGTAATCAAACC -3'	EMSA
*desA2*_ P_-36/+14_R_C	5'- GGTTTGATTACCATGAGGTAGTATA CCTTCGTATTTGCATCGTTCTCGAA-3'	EMSA
*hsp60*_P_F	5'-GGGCCGAACATACTCACCCG-3'	EMSA
*hsp60*_P_R	5'-TGCGAAGTGATTCCTCCGG-3'	EMSA

*desA1*_P_XbaI	5'-TTTTCTAGAGGTCTGTTCACCGCATATCT-3'	pSD5B LacZ transcriptional fusion constructs
*desA1*_P_SphI	5'-TTTGCATGCTTGTGCATGCTCAG-3'	pSD5B LacZ transcriptional fusion constructs LacZ transcriptional fusions
*desA2*_P_XbaI	5'-TTTTCTAGAGGGCCTGCGGATAGG-3'	pSD5B LacZ transcriptional fusion constructs
*desA2*_P_SphI	5'-TTAGCATGCGAACGGGACGTAG-3'	pSD5B LacZ transcriptional fusion constructs

Table S2. Genes significantly induced in Δ*madR* cells compared to WT and complement.

Gene ID	Product	Fold change (log2)	*P*-value
MSMEG_6451	transcriptional regulator, ArsR family protein	3.32	0.00981
MSMEG_5773	fatty acid desaturase	3.05	8.4E-130
MSMEG_1770	conserved hypothetical protein	2.95	3.9E-140
MSMEG_5774	tRNA-dihydrouridine synthase, putative	2.57	3.9E-94
MSMEG_3757	ribosomal RNA	2.57	4.7E-10
MSMEG_4931	ribosomal RNA	2.55	4.9E-10
MSMEG_5757	tRNA	2.46	1.3E-08
MSMEG_5248	acyl-[ACP] desaturase	2.22	2.5E-128
MSMEG_3932	14 kDa antigen	2.01	1.9E-08
MSMEG_1338	tRNA	1.67	6.0E-16
MSMEG_4929	ribosomal RNA	1.59	7.3E-08
MSMEG_4930	ribosomal RNA	1.50	2.9E-06
MSMEG_3937	conserved hypothetical protein	1.47	0.00534
MSMEG_3756	ribosomal RNA	1.47	3.3E-06
MSMEG_5247	PhoH family protein	1.46	9.2E-70
MSMEG_1337	tRNA	1.45	0.00069
MSMEG_3755	ribosomal RNA	1.41	3.6E-08
MSMEG_0532	transcriptional regulator, TetR family protein	1.40	8.9E-12
MSMEG_4892	conserved hypothetical protein	1.38	6.4E-13
MSMEG_3934	phosphoenolpyruvate synthase	1.36	5.4E-06
MSMEG_4645	alpha oxoglutarate ferredoxin oxidoreductase, beta subunit	1.34	6.1E-56
MSMEG_3928	[NiFe] hydrogenase, alpha subunit, putative	1.33	0.00026
MSMEG_2027	conserved hypothetical protein TIGR00026	1.33	9.4E-14
MSMEG_3946	probable conserved transmembrane protein	1.33	0.00037
MSMEG_4326	acyl carrier protein	1.31	1.9E-35
MSMEG_1905	acyl-CoA dehydrogenase protein	1.28	0.00015
MSMEG_1783	hypothetical protein	1.27	9.9E-21
MSMEG_5016	conserved domain protein	1.25	1.8E-05
MSMEG_4604	hypothetical protein	1.23	0.00320
MSMEG_4056	conserved secreted protein	1.16	0.00672
MSMEG_1489	conserved hypothetical protein	1.14	0.00510
MSMEG_1076	conserved hypothetical protein	1.11	2.0E-25
MSMEG_4646	pyruvate synthase	1.11	1.7E-45
MSMEG_1194	hypothetical protein	1.10	1.2E-05
MSMEG_4747	hypothetical protein	1.09	4.5E-05
MSMEG_0180	putative HTH-type transcriptional regulator	1.08	0.00085
MSMEG_3955	conserved hypothetical protein	1.08	0.00038
MSMEG_3931	[NiFe] hydrogenase, beta subunit, putative	1.06	0.00014
MSMEG_0399	conserved domain protein	1.01	9.0E-21

MSMEG_1751	norsolorinic acid reductase	1.01	0.00053
MSMEG_3469	TetR family protein transcriptional regulator	1.00	0.00027
MSMEG_4706	tRNA	-2.85	0.00794
MSMEG_5111	conserved hypothetical protein	-2.42	4.6E-05
MSMEG_0173	hypothetical protein	-2.12	0.00057
MSMEG_5539	hypothetical protein	-1.54	0.00075
MSMEG_5234	short-chain dehydrogenase/reductase SDR	-1.49	0.00042
MSMEG_1220	ABC-type transport system permease protein I	-1.48	3.5E-05
MSMEG_6455	conserved hypothetical protein	-1.36	5.4E-06
MSMEG_0245	probable conserved transmembrane protein	-1.36	0.00035
MSMEG_6691	glutamine amidotransferase	-1.35	0.00046
MSMEG_1428	glucose-methanol-choline oxidoreductase	-1.33	9.1E-08
MSMEG_6437	copper resistance protein D	-1.30	0.00025
MSMEG_0626	conserved hypothetical protein	-1.29	0.00020
MSMEG_6637	hypothetical protein	-1.28	0.00033
MSMEG_0323	acyl-CoA dehydrogenase, short-chain specific, putative	-1.25	0.00452
MSMEG_4666	myo-inositol 2-dehydrogenase	-1.25	0.00111
MSMEG_5165	integral membrane protein	-1.25	0.00578
MSMEG_0554	ABC transporter permease protein, putative	-1.24	0.00846
MSMEG_5902	conserved hypothetical protein	-1.23	1.9E-09
MSMEG_0296	transcriptional regulator, MarR family protein	-1.23	0.00297
MSMEG_5885	short chain dehydrogenase	-1.21	0.00088
MSMEG_6635	alpha subunit of malonate decarboxylase	-1.19	1.4E-06
MSMEG_6925	conserved hypothetical protein	-1.18	0.00020
MSMEG_0729	conserved hypothetical protein	-1.18	0.00089
MSMEG_4585	ribokinase	-1.18	0.00132
MSMEG_3786	D-amino acid deaminase	-1.18	0.00039
MSMEG_1422	conserved hypothetical protein	-1.17	2.9E-08
MSMEG_6689	CAIB/BAIF family protein	-1.17	0.00137
MSMEG_5287	dehydrogenase	-1.16	3.9E-05
MSMEG_6585	acyl-CoA dehydrogenase	-1.16	7.6E-05
MSMEG_0438	Periplasmic binding protein	-1.16	7.0E-07
MSMEG_6072	Citrate transporter	-1.15	0.00039
MSMEG_5045	D-2-hydroxyglutarate dehydrogenase	-1.15	0.00183
MSMEG_6798	ribose operon repressor	-1.15	0.00100
MSMEG_0549	ABC transporter, permease protein	-1.14	6.3E-08
MSMEG_1239	ISMsm4, transposase	-1.14	1.9E-06
MSMEG_5791	conserved hypothetical protein	-1.14	0.00045
MSMEG_0999	integral membrane protein	-1.13	0.00015
MSMEG_5147	sugar ABC-transporter integral membrane protein	-1.13	0.00153
MSMEG_1421	hypothetical protein	-1.13	0.00010
MSMEG_5593	pyruvate dehydrogenase	-1.12	5.8E-06
MSMEG_6033	hypothetical protein	-1.12	1.2E-05
MSMEG_4469	cobalt transport protein	-1.11	4.2E-05

MSMEG_6456	conserved hypothetical protein	-1.11	0.00029
MSMEG_6011	short chain dehydrogenase	-1.11	0.00396
MSMEG_5794	hypothetical protein	-1.11	0.00010
MSMEG_1067	conserved hypothetical protein	-1.10	0.00096
MSMEG_5980	methyltransferase	-1.10	0.00154
MSMEG_5437	probable serine/threonine-protein kinase PknB	-1.10	0.00359
MSMEG_0156	transcriptional regulator, LysR family protein	-1.10	0.00281
MSMEG_2650	acyl-CoA dehydrogenase	-1.09	9.5E-07
MSMEG_0776	conserved hypothetical protein	-1.09	0.00473
MSMEG_0812	amino acid transporter	-1.09	0.00290
MSMEG_4255	conserved hypothetical protein	-1.09	0.00022
MSMEG_2610	cobalt transport protein ATP-binding subunit	-1.09	0.00198
MSMEG_0625	conserved hypothetical protein	-1.08	0.00200
MSMEG_5629	conserved hypothetical protein	-1.08	0.00130
MSMEG_5717	pyridoxamine 5'-phosphate oxidase family protein	-1.08	0.00036
MSMEG_5279	conserved hypothetical protein	-1.07	0.00075
MSMEG_6803	ribose transport system permease protein RbsC	-1.06	5.7E-05
MSMEG_4565	oxidoreductase	-1.06	0.00587
MSMEG_3916	hypothetical protein	-1.05	0.00373
MSMEG_0833	conserved hypothetical protein	-1.05	1.4E-07
MSMEG_5930	conserved hypothetical protein	-1.05	0.00160
MSMEG_0743	xanthine dehydrogenase	-1.04	0.00036
MSMEG_2592	conserved hypothetical protein	-1.04	0.00160
MSMEG_0032	cell cycle protein, FtsW/RodA/SpoVE family protein	-1.04	2.4E-09
MSMEG_0595	glycolate oxidase	-1.03	4.0E-06
MSMEG_0991	NAD-dependent epimerase/dehydratase	-1.03	6.8E-05
MSMEG_1452	sulfatase-modifying factor 1	-1.03	0.00022
MSMEG_0443	hydrolase, carbon-nitrogen family protein	-1.03	0.00129
MSMEG_4982	conserved hypothetical protein	-1.02	0.00708
MSMEG_6046	cation ABC transporter, ATP-binding protein, putative	-1.02	0.00095
MSMEG_5577	fructokinase	-1.02	0.00111
MSMEG_0068	probable conserved transmembrane protein	-1.02	2.0E-11
MSMEG_0993	histidine kinase	-1.02	0.00127
MSMEG_1741	TetR-family protein transcriptional regulator	-1.02	0.00056
MSMEG_0623	secretion protein Snm4	-1.01	0.00079
MSMEG_4962	RemO protein	-1.01	0.00062
MSMEG_6686	glutaryl-CoA dehydrogenase	-1.01	0.00030
MSMEG_2227	carnitinyl-CoA dehydratase	-1.01	0.00919
MSMEG_6523	ABC transporter, membrane spanning protein	-1.01	0.00070
MSMEG_5051	major facilitator superfamily protein	-1.01	3.3E-06
MSMEG_4657	ABC transporter membrane protein	-1.01	0.00110
MSMEG_0439	conserved hypothetical protein	-1.00	0.00054
MSMEG_6390	transporter, major facilitator family protein	-1.00	0.00163
